# Supplementary material for: GTP biosynthesis is a therapeutic vulnerability in Rac1-mutant melanoma
Source: bioRxiv. 2026 Sep 24:2026.09.23.753957. Preprint. [Version 1] doi: 10.64898/2026.09.23.753957 (PMC13622192; doi:10.64898/2026.09.23.753957)
Supplement: Supplementary file 1 [file media-1.pdf]

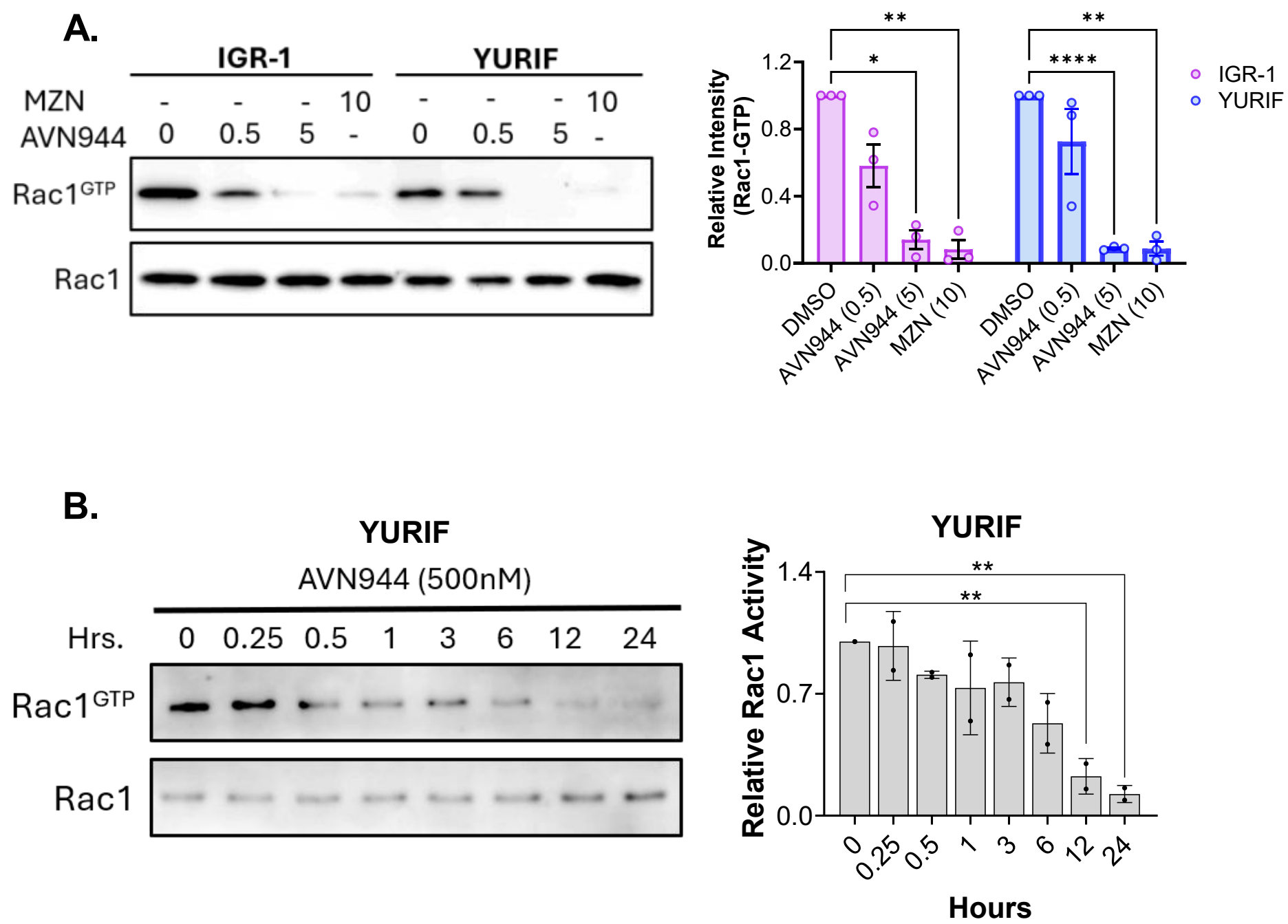

Supplemental Figure S1

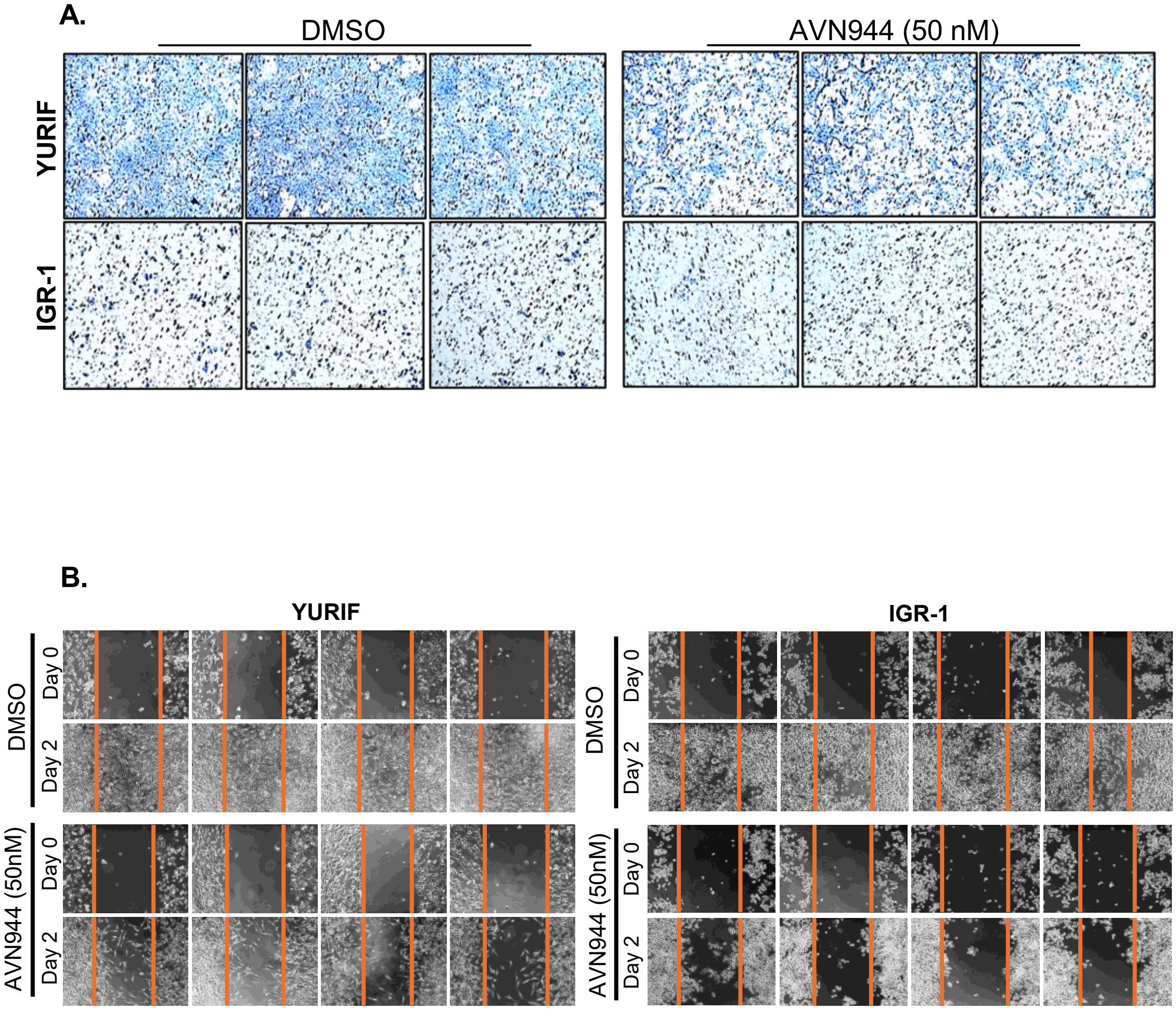

Supplemental Figure S2

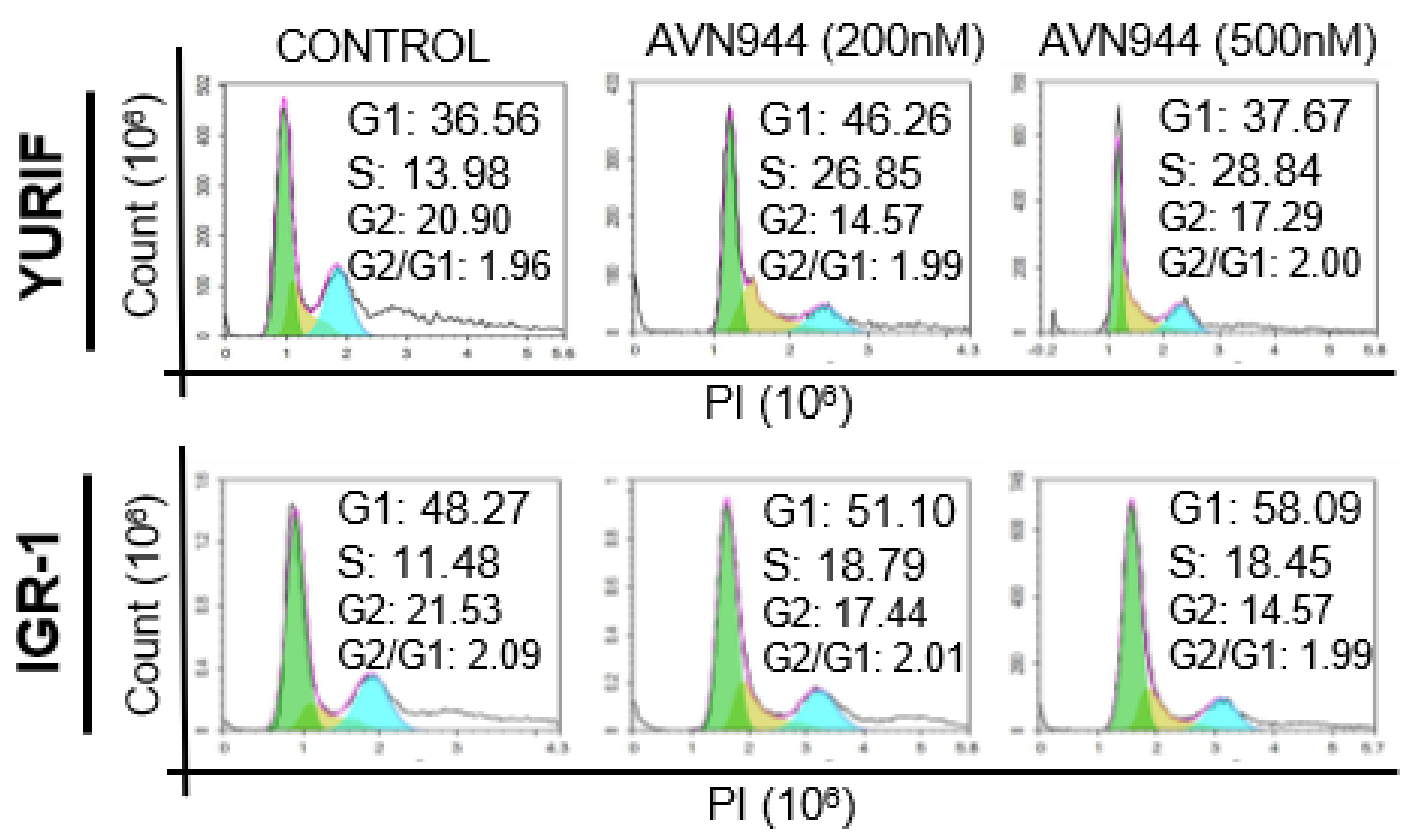

Supplemental Figure S3

A.

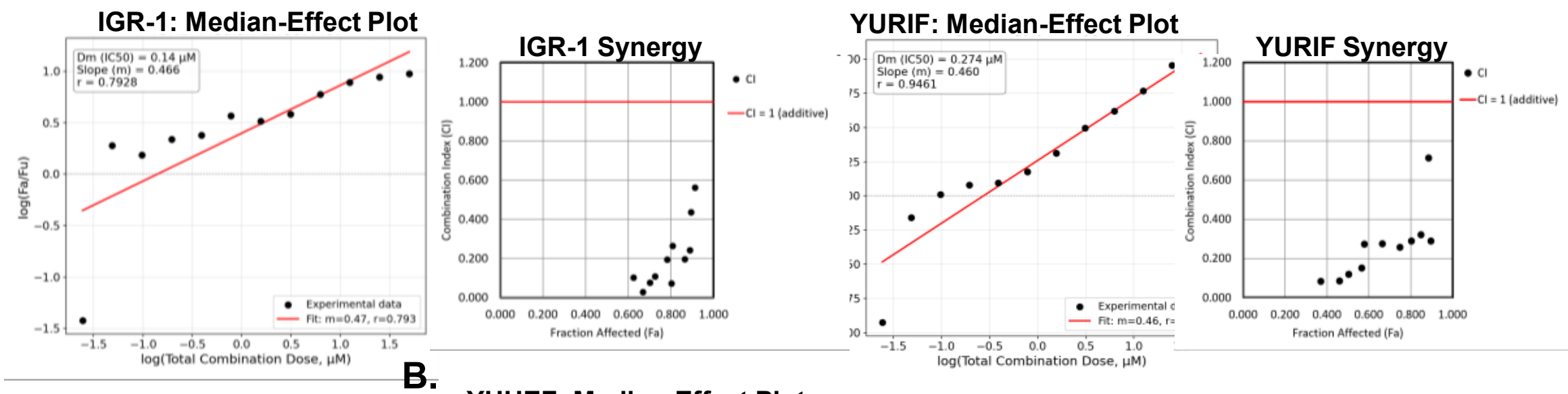

B.

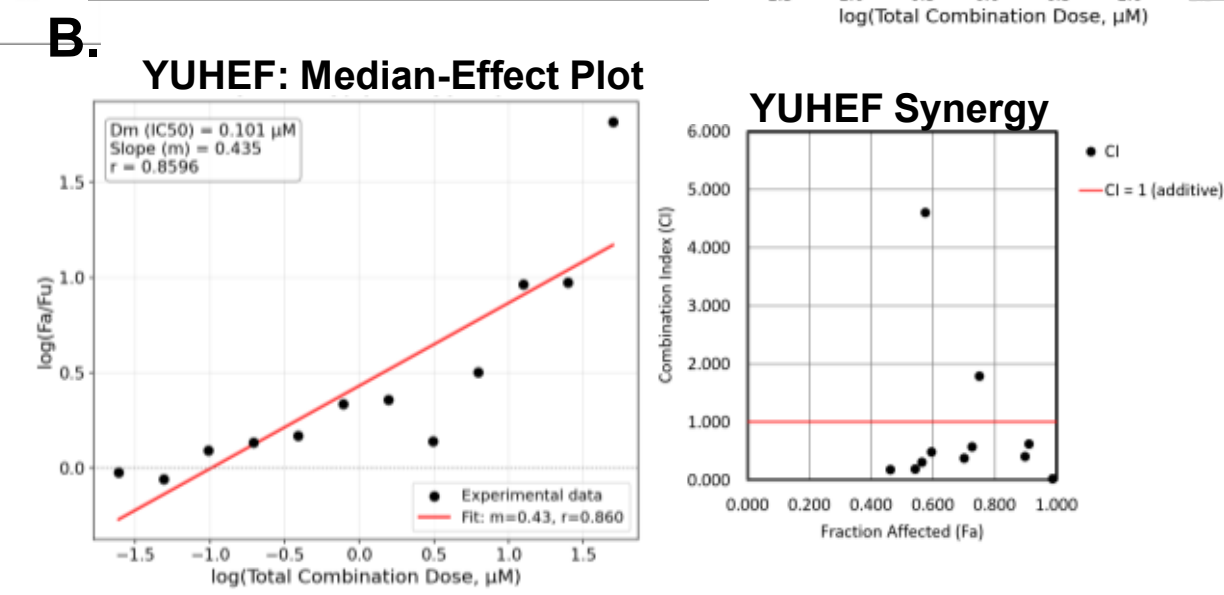

Supplemental Figure S4

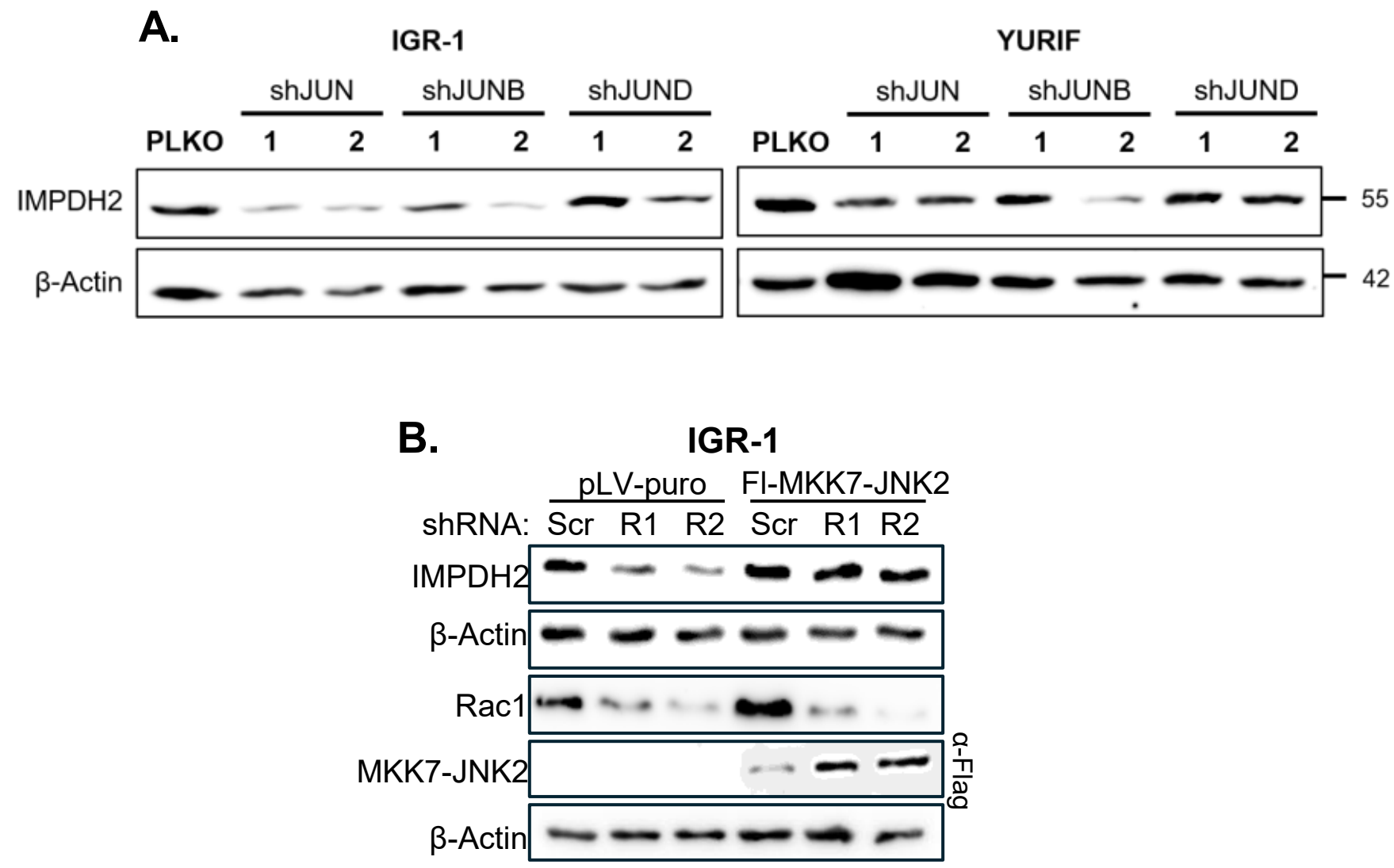

Supplemental Figure S5
